# Hypothalamic and pituitary transcriptome profiling using RNA-sequencing in high-yielding and low-yielding laying hens

**DOI:** 10.1038/s41598-019-46807-3

**Published:** 2019-07-16

**Authors:** Chunqiang Wang, Wei Ma

**Affiliations:** College of Animal Science and Veterinary Medicine, Jinzhou Medical University, Jinzhou, Liaoning 121001 P.R. China

**Keywords:** Sequencing, Gene expression

## Abstract

The reproductive physiology and laying performance of laying hens are regulated by the hypothalamus and pituitary. To understand the mechanism of egg laying regulation, we sequenced and analysed the hypothalamus and pituitary expression profiles in high- and low-yielding laying Chinese Dagu Chickens (CDC) using RNA-seq. More than 46 million clean reads and 24,873 tentative genes were obtained using the *Gallus gallus* genome as a reference. Transcriptome analysis in hypothalamus and pituitary revealed seven and 39 differentially expressed genes (DEGs) between high- and low-yielding CDC hens, respectively. A total of 24 and 22 DEGs were up-regulated and down-regulated, respectively, and 13 novel genes were identified. Functional annotation and pathway enrichment analysis showed that DEGs in the hypothalamus were mainly enriched in glycosaminoglycan biosynthesis. DEGs significantly enriched in the pituitary primarily affected the extracellular matrix, the protein extracellular matrix, and the extracellular space. Pathways involving phenylalanine metabolism, 2-oxocarboxylic acid metabolism, the glycosphingolipid biosynthesis-ganglion series, and local adhesion were significantly enriched in the pituitary. Eight DEGs, *PRDX6*, *TRIB2*, *OVCH2*, *CFD*, *Peptidase M20*, *SLC7A10*, and two other amino acid transporters, are involved in the metabolism and transport of amino acids. To our knowledge, this is the first study comparing the hypothalamus and pituitary transcriptomes of high- and low-yielding laying hens. Our findings suggest that putative differences in gene expression can provide a base for further research in this field. Moreover, we identified increased expression of genes involved in amino acid metabolism, glycosaminoglycan biosynthesis, and oestrogen negative feedback systems in low-yielding laying hens, highlighting their potential as biomarkers of egg production.

## Introduction

Chinese Dagu Chickens (CDC) are a local variety distributed in Liaoning Province, China. They are famous for their large bodies, quality eggs, delicious meat, and strong disease resistance. However, lack of systematic selection and breeding in the commercial production of CDC laying hens, means that the individual performance of egg production within the population varies. The selection of partial, or annual, egg counts or egg production rates is a common method for improving the laying performance of CDC hens. This can produce some positive genetic progress but it is slow^1,2^.

Increasing egg yield is one of the main purposes of breeding in laying hens^3^. However, most of the chicken laying traits, including egg production, egg weight, and age of sexual maturity, are polygenic traits with low to moderate heritability, making it difficult to estimate the level of genetic improvement in each generation^4^. Recently, RNA-sequencing (RNA-seq), a next-generation sequencing method, has become a commonly used technique for transcriptome analysis and the exploration of unknown genes^5^. To date, RNA-seq has been used for high-throughput genetic analysis of a variety of livestock, including cattle^6^, sheep^7^, and goats^8^.

Studies of the regulation mechanism of egg laying in hens have focused on changes in gene regulation during different follicular stages^9,10^. However, like in vertebrates, poultry reproductive activity is controlled by the hypothalamic-pituitary-gonadal axis, which has been overlooked in studies of egg laying^11,12^. Changes in the function of the hypothalamus or pituitary, either alone or simultaneously, will affect reproductive activities, including follicular development, ovulation, and spawning^13^. Therefore, the hypothalamus and pituitary gland should be sources of molecular markers associated with the ideal tissue for egg production.

This study aims to compare and analyse the expression profiles of the hypothalamus and pituitary in high- and low-yielding laying CDC hens using RNA-seq. The gene expression patterns and new genes identified in this study will highlight the important genes involved in regulating the biological processes of egg production. Genes discovered through this study will have potential as molecular markers for genetic selection applications in laying hen breeding programs.

## Results

### Summary of the raw sequence reads

A total of 12 cDNA libraries were constructed from the hypothalamus and pituitary of high- and low-yielding laying hens, and the raw reads of each library is more than 49 million. After filtering to remove low-quality and linker sequences, more than 46 million clean reads remained. Among these clean reads, more than 96.18 and 90.76% had quality scores at the Q20 and Q30 levels, respectively. The clean reads had GC contents between 51.60 and 53.57% and 85.57 to 87.92% of sequences were mapped to the genome. The read distribution was determined, and the read density on the chromosomes showed that the read content was highest on exons, and accounted for 62.1 to 70.7% of reads (S 1).The fastq files have been submitted to Sequence Read Archive database (SRA accession PRJNA551380).

### Differentially expressed genes

**Figure 1:**
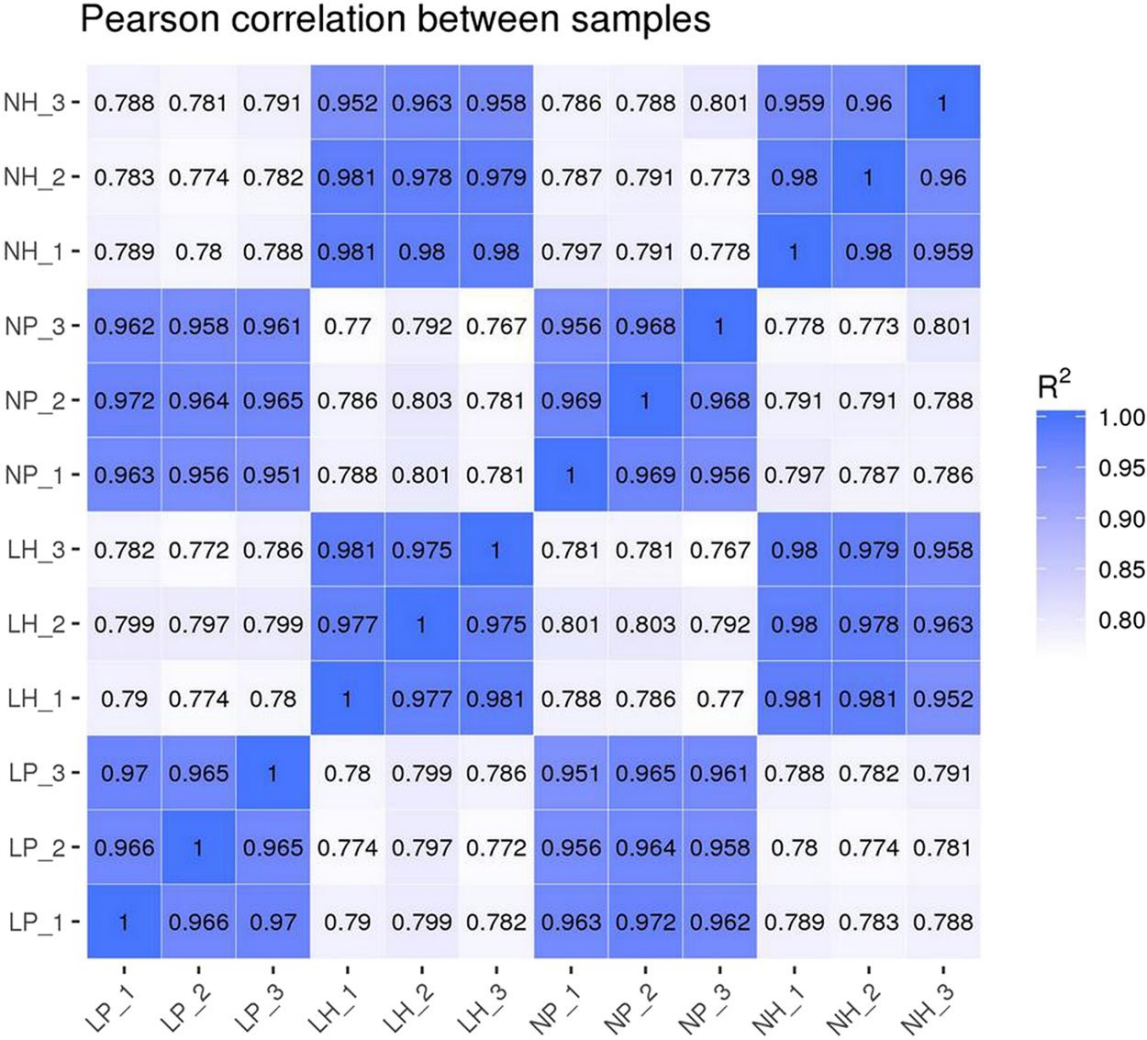
RNA-Seq correlation check. LP_1 ∼ LP_3 are pituitary samples of high-yielding laying hens from 1 to 3, and NP_1 ∼ NP_3 are pituitary samples of low-yielding laying hens from 1 to 3. LH_1 ∼ LH_3 are hypothalamic sample of high-yielding laying hens from 1 to 3, and NH_1 ∼ NH_3 are hypothalamic sample of low-yielding laying hens from 1 to 3.The horizontal and vertical coordinates are log10 (FPKM + 1) of the samples compared with each other.

**Figure 2:**
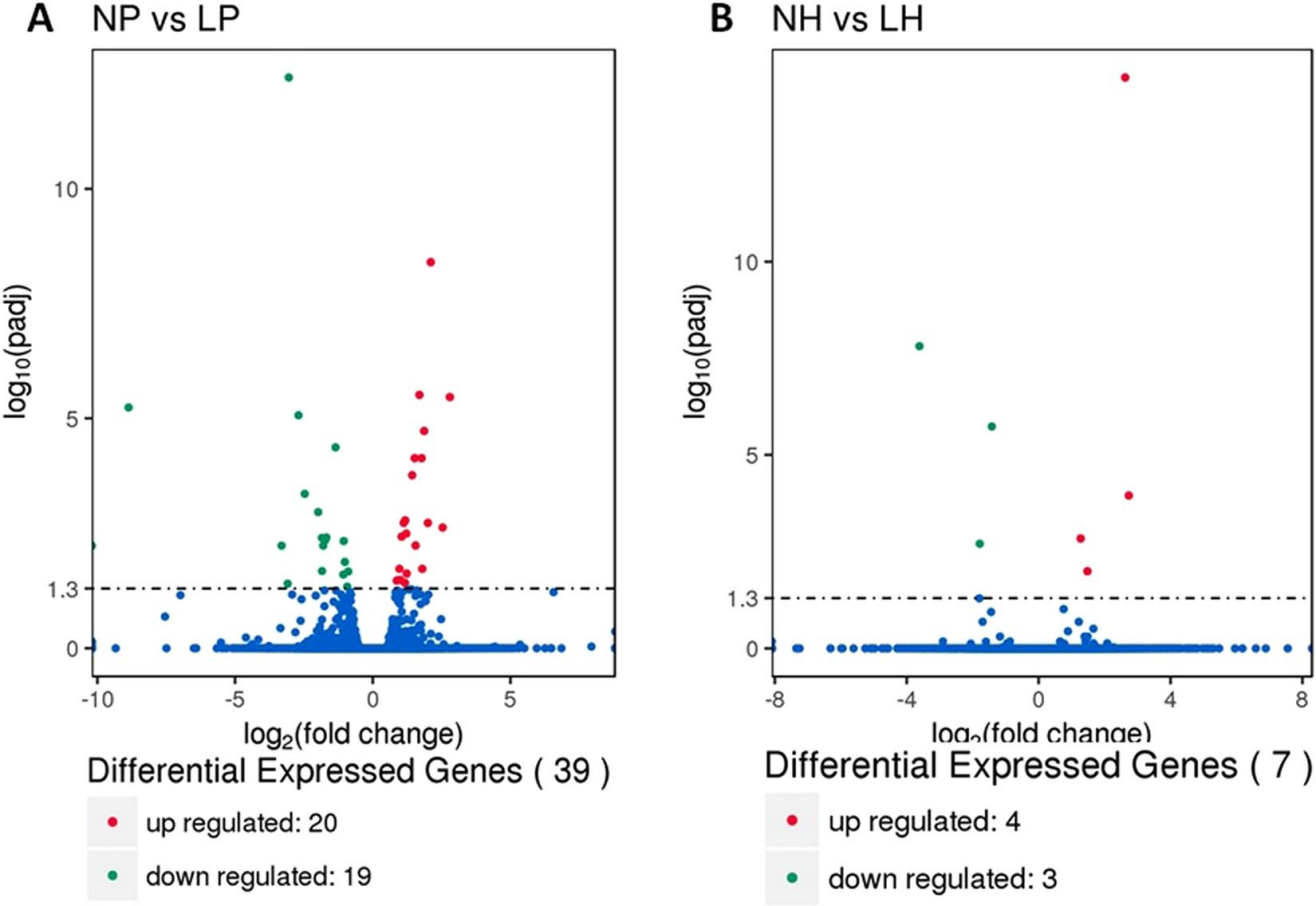
Volcanic map of differentially expressed genes. (**A**) LP and NP represent pituitary samples of high-yielding laying hens and low-yielding laying hens respectively. (**B**) LH and NH represent hypothalamic samples of high-yielding laying hens and low-yielding laying hens respectively. The abscissa represents the fold change in the expression of the gene in different samples; The ordinate represents the statistical significance of the difference in the amount of gene expression; The red dot in the figure indicates the up-regulated gene with significant differential expression, and the green dot indicates the down-regulated gene with significant differential expression.

**Figure 3:**
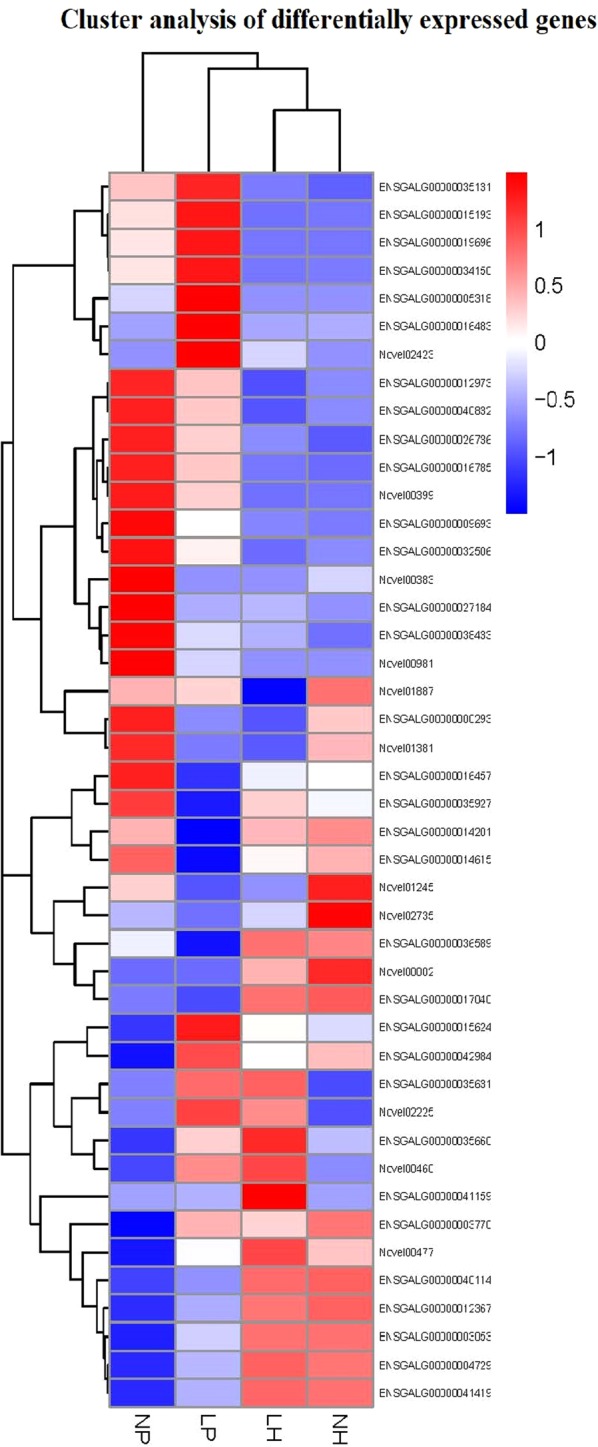
Cluster Analysis of Differentially Expressed Genes. LP and NP represent pituitary samples of high-yielding laying hens and low-yielding laying hens respectively. LH and NH represent hypothalamic samples of high-yielding laying hens and low-yielding laying hens respectively; The red blocks represent the overexpressed genes, and the blue blocks represent genes with the lowest expression levels. Colored bars indicate the expression levels.

Gene expression levels were quantified by FPKM^14^. The correlation coefficient of gene expression levels between samples was higher than 0.952, indicating that the selection of experimental samples was consistent and reliable (Fig. 1). A total of 39 DEGs were identified in the six pituitary cDNA libraries, including 20 up-regulated genes and 19 down-regulated genes in low-yielding hens. In the six hypothalamic cDNA libraries, seven DEGs were found, of which four were up-regulated and three were down-regulated in low-yielding hens (Fig. 2A,B). Some representative genes are listed in Table 1. The FPKM hierarchical clustering map (Fig. 3) visually reflects the expression patterns of the genes in the samples and highlights the reproducibility and credibility of the data. We found two common DEGs in the pituitary and hypothalamus of the chicken.

**Table 1:** List of partially representative DEGs.

Organization	Different expression type	Genes	Description	log_2_FoldChange	Pvalue
Hypothalamus	Up-regulated	Pax-4	paired box protein Pax-4	1.480	2.92E-06
	Down-regulated	MANBAL1	mannosidase, beta A, lysosomal-like 1	−1.419	2.29E-10
		XYLT2L	xylosyltransferase 2-like	−3.613	1.28E-12
Pituitary	Up-regulated	GFRA4	Glial cell line-derived neurotrophic factor receptor	2.104	3.34E-13
		LAMA1	Laminin EGF domain	1.865	5.56E-09
		CFD	Peptidase S1, PA clan	1.695	3.90E-10
		OLFML1	Olfactomedin-like protein 1	1.556	5.84E-06
		ESR1	Zinc finger, nuclear hormone receptor-type	0.969	2.35E-05
	Down-regulated	−	Amino acid/polyamine transporter I	−8.868	1.22E-09
		OVCH2	Serine proteases	−3.312	6.41E-06
		VCAN	Immunoglobulin subtype	−3.091	6.29E-05
		CATHL2	Cathelicidin	−2.698	2.17E-09
		SLC7A10	Amino acid/polyamine transporter I	−1.843	2.71E-05

### GO enrichment analysis

**Figure 4:**
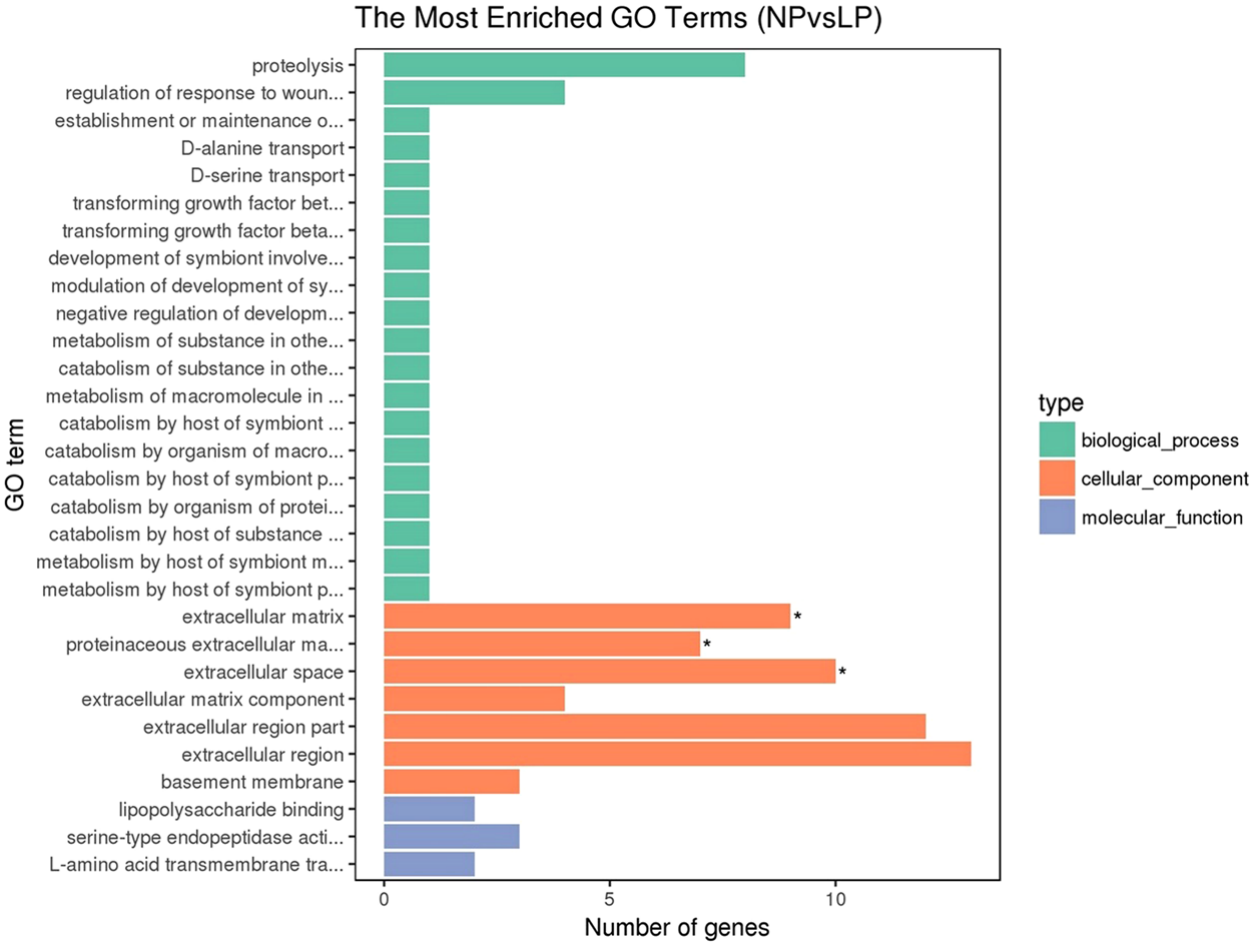
GO-enriched DAG map of differentially expressed genes. LP and NP represent pituitary samples of high-yielding laying hens and low-yielding laying hens respectively. Green indicates biological processes; orange indicates cellular components; blue indicates molecular function.

To further elucidate the function of DEGs, we used GO enrichment analysis to annotate DEGs and to study their distribution. In the LP (high-yielding hens’ pituitary) and NP (low-yielding hens’ pituitary) comparison groups, most DEGs were involved in biological processes, followed by cellular components, and molecular functions. Among them, 12 DEGs were significantly enriched in the ‘cell component’ term (corrected p-value < 0.05). These significantly enriched DEGs primarily affect the extracellular matrix, the protein extracellular matrix, and the extracellular space (Fig. 4).

### KEGG pathway analysis

**Figure 5:**
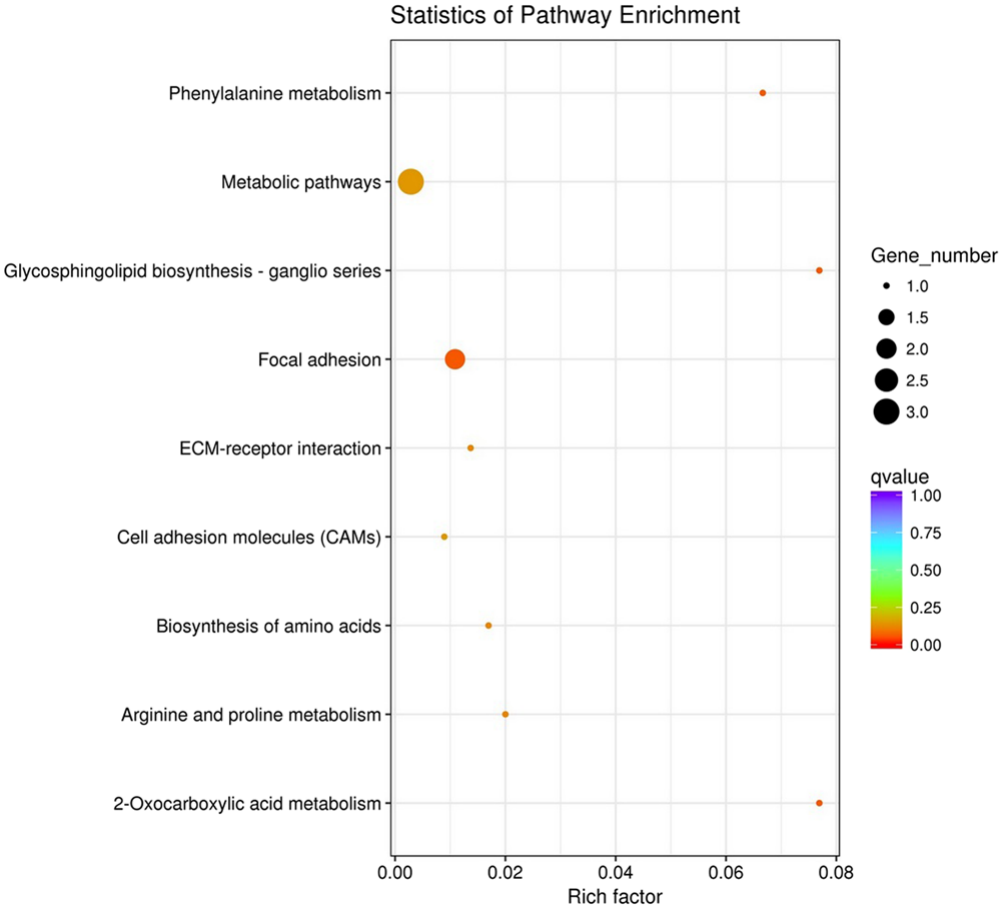
KEGG enrichment scatter plot of differentially expressed genes. LP and NP represent pituitary samples of high-yielding laying hens and low-yielding laying hens respectively. The ordinate indicates the path name, and the abscissa indicates the Rich factor. The size of the point indicates how many differentially expressed genes are in the pathway, and the color of the point corresponds to a different Q-value range.

To further identify the major biochemical, metabolic, and signal transduction pathways of the DEGs, we performed a KEGG pathway enrichment analysis. In the pituitary, comparison of NP and LP, yielded a total of 12 genes mapped to nine KEGG pathways, four of which were significantly enriched (corrected p-value < 0.05). These pathways involved 2-oxocarboxylic acid metabolism, glycosphingolipid biosynthesis-ganglion series, phenylalanine metabolism, and the local adhesion pathways. Five DEGs were associated with these pathways, and the local adhesion pathway was associated with two DEGs (Fig. 5).

### Protein interaction network analysis

**Figure 6:**
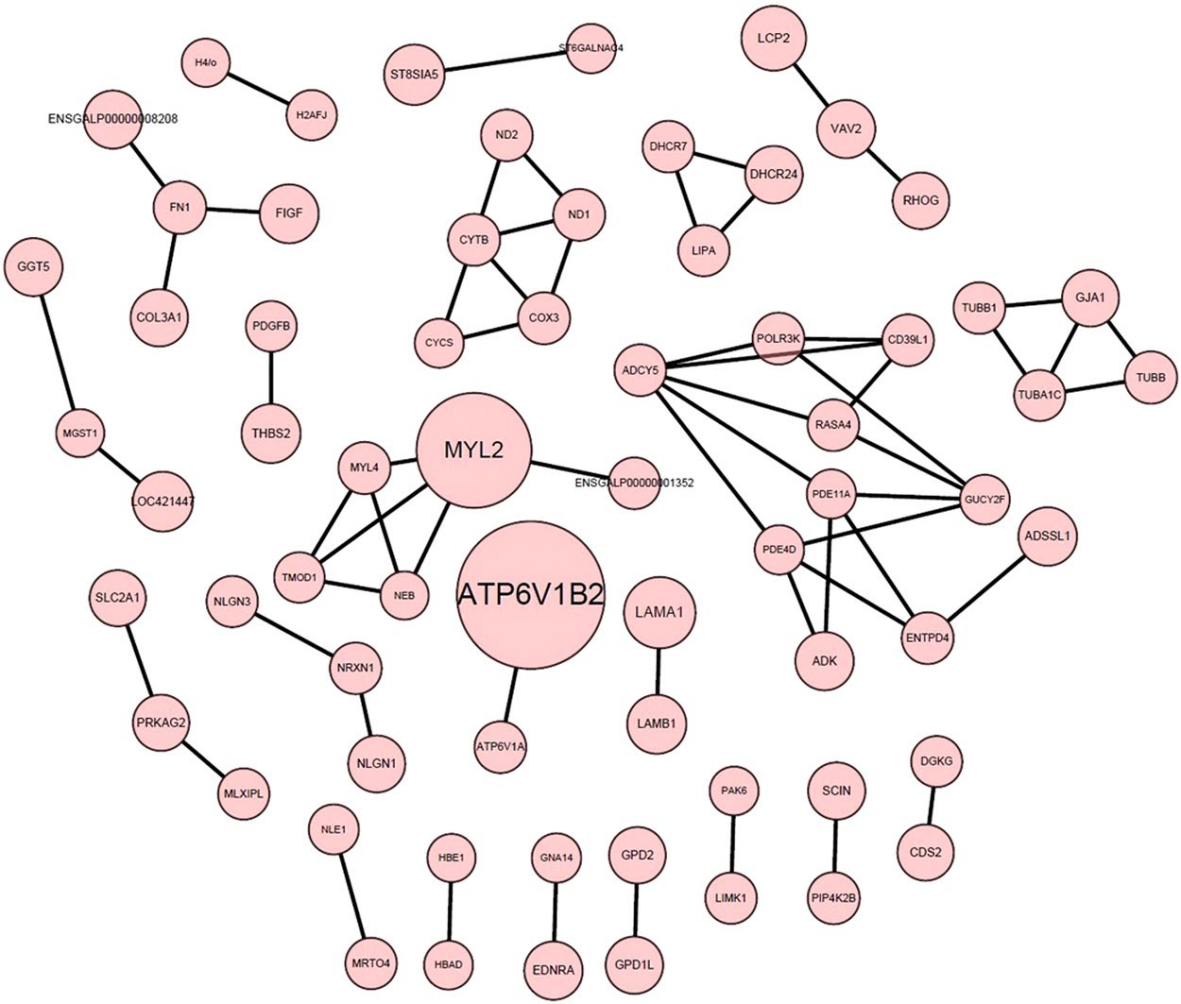
Map of protein-protein interaction networks in pituitary. The node size represents the expression of DEGs, the greater the node size, the greater the upward regulate.

The relationship between the proteins encoded by the DEGs was further analysed. The parameters were set to p-val < 0.05, fc ≥ 1, and a schematic of the protein-protein interaction network is shown in Fig. 6. In the pituitary, there was an interaction between the 93 proteins. MYL2, CYTB, and PDE11A, which are mainly involved in amino acid metabolism, ATP synthesis, actin filament formation, and purinergic neurotransmission, may be in the central regulatory position of the interaction network.

### Verification of DEGs by qRT-PCR

**Figure 7:**
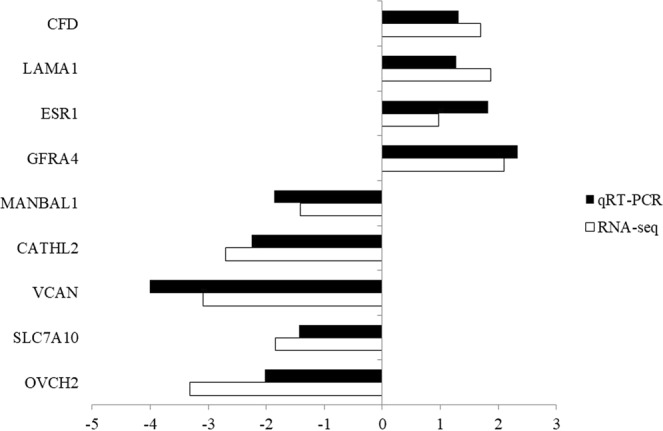
qRT-PCR for nine candidate genes. The relative expression level of each gene was calculated by the 2^−ΔΔCT^ method, and *beta-actin* was used as the internal control for normalization of the results.

Nine candidate genes were selected for qRT-PCR analysis. These included four genes that were up-regulated in the pituitary (*CFD*, *LAMA1*, *ESR1*, and *GFRA4*), four genes that were down-regulated in the pituitary (*CATHL2*, *VCAN*, *SLC7A10*, and *OVCH2*), and one gene that was down-regulated in the hypothalamus (*MANBAL1*). While there are clear differences in the expression levels of these genes, the observed expression trends confirmed the RNA-seq results (Fig. 7), indicating that the RNA-seq results are reliable.

## Discussion

The hormones and factors produced by the hypothalamus and pituitary of birds act on target organs including the gonads, to maintain normal reproductive functions. These factors are responsible for the initiation of sexual maturation, seasonal gland development, and avian reproductive behaviour^15,16^. Studies have shown that candidate genes such as prolactin (*PRL*) and GTPase-activating Rap-GAP domain-like 1 (*GARNL1*) in the pituitary may affect the laying rate of laying hens. However, this notion is based on the selection of specific candidate genes, and it is impossible to objectively and accurately identify genes of the hypothalamus and pituitary that are involved in the laying process of chickens^17^. The secretion of reproductive hormones in the hypothalamic-pituitary-ovarian axis (HPOA) is affected by many factors. To minimize the influence of other factors on the gene expression profile of the hypothalamus and pituitary, a criterion for selecting hens is that each hen does not produce soft shell eggs. Studies have shown that hormones such as the luteinizing hormone (LH) and progesterone (P) fluctuate greatly in hens producing soft shell eggs. The second standard of hens is that the laying period is short, i.e., 11 am every day, and the deviation around this time point is no more than 20 min. Thus, laying hens were selected based on these criteria and the hypothalamus and pituitary gland were taken uniformly at 8 am. Here, transcriptome analysis of the pituitary in high- and low-yielding laying CDC hens identified 39 DEGs. Of these DEGs, 20 were up-regulated, 19 were down-regulated, and nine were novel genes. Twelve DEGs, including *laminin alpha and domain I* (*LAMA1*), were significantly enriched and mapped to extracellular matrices, proteinaceous extracellular matrices, and extracellular spaces. The extracellular matrix is a three-dimensional network structure composed of proteins, glycosaminoglycans, and other macromolecules^18^ and plays an important role in the function of animal endocrine systems. The extracellular matrix controls hormone secretion in different glands, including regulating testosterone production in Leydig cells and promoting the synthesis and secretion of follicle stimulating hormone receptor and progesterone in follicular granulosa cells^19,20^. Here, transcriptome analysis of the pituitary in high- and low-yielding laying CDC hens identified 39 DEGs. Of these DEGs, 20 were up-regulated, 19 were down-regulated, and nine were novel genes. Twelve DEGs, including *laminin alpha and domain I* (*LAMA1*), were significantly enriched and mapped to extracellular matrices, proteinaceous extracellular matrices, and extracellular spaces. The extracellular matrix is a three-dimensional network structure composed of proteins, glycosaminoglycans, and other macromolecules^19^ and plays an important role in the function of animal endocrine systems. The extracellular matrix controls hormone secretion in different glands, including regulating testosterone production in Leydig cells and promoting the synthesis and secretion of follicle stimulating hormone receptor and progesterone in follicular granulosa cells^20,21^.

KEGG pathway enrichment analysis of DEGs in high- and low-yielding hen pituitary identified a total of 12 DEGs that mapped to nine KEGG pathways. 2-oxocarboxylic acid metabolism, glycosphingolipid biosynthesis, phenylalanine metabolism, and local adhesion pathways were significantly enriched. Three of these pathways are involved in amino acid biosynthesis and metabolism. DEGs involved include *Carboxypeptidase X Member 2* (*CPXM2*), *peroxiredoxin AhpC* (*PRDX6*), *peptidase M20*, *tribbles homolog 2* (*TRIB2*), *ovochymase 2* (*OVCH2*) and *complement factor D* (*CFD*). *CPXM2* was found to be involved in cellular processes of early spermatogonia development in chicken^22^. *OVCH2* plays an important role in egg production and ovulation in the sea squirt, but there are no reports of such function in mammals or poultry^23^. It has been reported that various serine proteases are involved in the sexual reproduction of sea squirt, including affecting egg production, oocyte maturation^24^, and affecting the fertilization process^25^. We therefore hypothesized that a significant decrease of *OVCH2* expression in pituitary may have a negative effect on the egg production, maturation, and ovulation of laying hens. *Solute carrier family 7 member 10* (*SLC7A10*) and two unannotated amino acid transporters were significantly down-regulated in the pituitary of low-yielding chickens. While there has been extensive research into the physiological functions of *SLC7A10* in mice and humans, its role in poultry has not been examined. SLC7A10 is a sodium-independent amino acid transporter that promotes the transport of many amino acids, including glycine, L-serine, L-alanine, and L-cysteine^26^. SLC7A10 is described as a neuronal transporter whose primary role is in the regulation of excitatory glutamatergic neurotransmission and the level of glycine in the brain of SLC7A10-deficient mice was significantly decreased. Decreased brain glycine is the main cause of motor neuron abnormalities and early postnatal mortality and is involved in various neurological disorders^27^.

Amino acids are biological activity regulators of physiological systems such as the reproductive, cardiovascular, and immune systems of animals^28^. L-arginine can alter pituitary function and increase blood flow to the reproductive tract, providing nutrients or other substances for follicular development and has important implications for gestational development in the uterus^29,30^. In gilts (young female pigs), oral supplementation with L-arginine from day 30 of pregnancy increases litter size and birth weight^31^. These results show that amino acid metabolism in the pituitary may play an important role in regulating the reproductive physiology of chickens. Therefore, we hypothesise that, in the pituitary, down-regulation of genes affecting amino acid synthesis, transport, and metabolism may lead to abnormal amino acid utilization. This may affect the development and function of downstream target organs through the endocrine vasculature, ultimately influencing the reproductive physiological state and laying performance of hens.

Pituitary DEGs identified here include many receptor genes, such as *oestrogen receptor 1* (*ESR1*), *glial cell line-derived neurotrophic factor receptor* (*GFRA4*), and *interleukin-1 receptor* (*IL1RL1*). Of these three genes, only *ESR1* has been reported to function in the pituitary. Oestrogen is essential for the development of animal follicles, and both oestrogen receptors have independent functions^32^. Oestrogen receptors (ERs) are ligand-activated transcription factors that, when bound in the cytoplasm, transfer to the nucleus, causing an ER-centric signalling process^33^. Both subtypes of oestrogen receptor, ESR1 and ESR2, are present in the pituitary but the receptor knockout model indicates that ESR1 is the major receptor for negative feedback of oestrogen^34^. Moreover, oestrogen can negatively regulate pituitary function and regulate reproductive activities in females^35^. In addition, ESR1 gene polymorphism is closely related to egg production traits in Chinese Dagu chickens^36^. After dissection, we found that there were large follicles on the ovaries of low-yielding laying hens. Since ER expression occurs following oestrogen stimulation, we speculate that disorganization of ovulation leads to the persistence of large follicles, and that oestrogen-mediated negative feedback regulates pituitary function, which then affects the laying performance of hens.

Hypothalamus transcriptome analysis in high- and low-yielding laying hens revealed seven DEGs, of which four were up-regulated and three were down-regulated. This group also included four novel genes. The results of GO and KEGG analysis of DEGs in the hypothalamus revealed no significant enrichment of DEGs in GO terminology. However, DEGs were mainly enriched in glycosaminoglycan biosynthesis in the KEGG pathway. Only two of the DEGs, *xylosyltransferase 2-like* (*XYLT2L*) and *mannosidase beta A lysosomal-like 1* (*MANBAL1*), were able to be predicted or had been previously annotated. *XYLT2L* was one of the first glycosyltransferases discovered and changes in XYLT activity levels in the body serve as biomarkers for diabetes, diseases associated with fibrosis, and/or extracellular matrix turnover^37^. However, the mechanism of action of XYLT has not been fully elucidated. Changes in XYLT activity or secretion can represent changes in the secretory activity of certain cell types, even as a by-product, which has certain clinical significance^38^. Therefore, we speculate that low-yielding hens may have defects in extracellular matrix turnover, similar to the results observed in the pituitary.

The hypothalamic-pituitary-ovarian axis is the main system regulating the reproductive physiology of hens^39^. Classical theory suggests that the hypothalamus secretes gonadotropin-releasing hormone (GnRH) and other signals that regulate the synthesis and release of pituitary luteinizing hormone (LH) and follicle stimulating hormone (FSH) into the blood, to control gonadal development and sex hormone production^40^. Oestrogen, progesterone, and other hormones produced by the developing gonads can act on the pituitary or hypothalamus through the endocrine system and regulate the synthesis and release of gonadotropin and neuropeptide hormones through negative feedback^41^. Here, the expression of GnRH in the hypothalamus of low yielding chickens did not change, which is similar to the RNA-seq results observed in the goose hypothalamus^42^. In addition, while the expression of hormones such as pituitary FSH and LH were down-regulated, the difference was not significant after correction. Meanwhile, *ESR2* expression was significantly up-regulated in the hypothalamus. Our findings are not completely consistent with those of previous reports, and suggest that the GnRH-FSH/LH pathway may not be the direct path of determining egg laying performance in chickens. These findings will complement the classical regulatory pathways in the hen hypothalamic-pituitary-ovarian axis.

In conclusion, we characterised and evaluated the hypothalamus and pituitary transcriptome and quantified the expression profiles in high- and low-yielding CDC hens. Our findings suggest that putative gene expression differences can be a base for further research on this topic. Moreover, because increased expression of genes involved in amino acid metabolism, glycosaminoglycan biosynthesis, and the oestrogen negative feedback system were identified in low-yielding laying hens we confirm their potential as egg production biomarkers.

## Materials and Methods

### Ethics statement

All methods and procedures were conducted in accordance with the relevant guidelines formulated by the Ministry of Agriculture of the People’s Republic of China. The Ethical approval for the present study was obtained from the Ethical Committee of Jinzhou Medical University (201805024).

### Experimental animals and tissue collection

The six CDC hens used in this study were obtained from the CDC Farm of Jinzhou Medical University. The selected hens were divided into high-yielding laying hens and low-yielding laying hens according to the number of eggs laid by hens at the peak of 39–42 weeks of laying. The high-yielding group and the low-yielding group produced 25 and 14 eggs respectively during this period. In order to reduce the intra-group error, the number of eggs laid by each hen in the group was the same. The hens were slaughtered and hypothalamic and pituitary tissues were isolated, rapidly frozen in liquid nitrogen, and stored at −80 °C until use.

### Construction libraries and sequencing

Total RNA was extracted from tissue samples using Trizol reagent according to the manufacturer’s instructions. The degree of RNA degradation was analysed by agarose gel electrophoresis and RNA purity was detected using a Nanodrop 2000 spectrophotometer. The RNA concentration was accurately quantified by Qubit 2.0; and RNA integrity was detected using the Agilent 2100 Bioanalyzer. Following sample testing, a total amount of 3 µg RNA per sample was used as input material for the RNA sample preparations. Sequencing libraries were generated using NEBNext® UltraTM RNA Library Prep Kit for Illumina® (NEB, USA) following manufacturer’s recommendations and index codes were added to attribute sequences to each sample. Briefly, mRNA was purified from total RNA using poly-T oligo-attached magnetic beads. Fragmentation was carried out using divalent cations under an elevated temperature in NEBNext First Strand Synthesis Reaction Buffer (5×). First strand cDNA was synthesized using a random hexamer primer and M-MuLV Reverse Transcriptase (RNase H-). Second strand cDNA synthesis was performed subsequently using DNA Polymerase I and RNase H. The remaining overhangs were blunt ended by exonuclease/polymerase activities. After adenylation of the 3′ ends of DNA fragments, NEBNext Adaptor with a hairpin loop structure was ligated to prepare the DNA for hybridization. cDNA fragments of 250–300 bp in length were selected by purifying the library fragments with the AMPure XP system (Beckman Coulter, Beverly, USA). Then, 3 µl of the USER enzyme (NEB, USA) was used with size-selected, adaptor-ligated cDNA at 37 °C for 15 min followed by 5 min at 95 °C before PCR. PCR was then performed with Phusion High-Fidelity DNA polymerase, Universal PCR primers and the Index (X) Primer. Finally, PCR products were purified (AMPure XP system) and the library quality was assessed on the Agilent Bioanalyzer 2100 system.

### RNA-Seq data analysis

The library preparations were sequenced on an Illumina HiSeq. 2500 platform.Quality control of the reads was performed by using in-house written scripts. Raw data of Fastq format were initially processed by in-house perl scripts. In this step, clean reads were obtained by removing reads containing adapter, poly-N, and low-quality reads from raw data. Q20, Q30 and GC content were calculated for the clean data. All downstream analyses were based on clean data with high quality. The PE 150 paired-end sequencing strategy was used in this study. The chicken’s genome sequence (90 version) was downloaded from genomewebsite (ftp://ftp.ensembl.org/pub/current_fasta/gallus_gallus/dna/Gallus_gallus.Gallus_gallus-5.0.dna.toplevel.fa.gz). Index of the reference genome was built using Hisat2 v2.0.5 and paired-end clean reads were aligned to the reference genome using Hisat2 v2.0.5. The gene expression level was estimated by the number of normalized fragments per kilogram of transcript per million fragments (FPKM) method. The differential expression analysis of the two groups was performed using the DESeq. 2 R package (1.16.1) based on the readcount data. The resulting P-values were adjusted using the Benjamini and Hochberg’s approach for controlling the false discovery rate. Genes with an adjusted P-value < 0.05 found by DESeq. 2 were assigned as differentially expressed.The high-yielding group was used as the control group to analyze the differentially expressed genes in the pituitary or hypothalamus of the low-yielding group.

### Gene ontology and KEGG pathway analysis

Gene ontology (GO) (http://www.geneontology.org/) enrichment analysis of differentially expressed genes was performed using GOseq based on Wallenius non-central hyper-geometric distribution^43^. This includes three parts: molecular function, biological process, and cellular component. A specific p-value was used to determine if a DEG is enriched in the GO. Usually, a p-value < 0.05 is indicative of enrichment. Pathway enrichment analysis was assessed using the KEGG (Kyoto Encyclopedia of Genes and Genomes) database^44^ (http://www.genome.jp/kegg/). The ClusterProfiler R package was used to test the statistical enrichment of differential expression genes in KEGG pathways. A p-value < 0.05 was considered indicative of the function being enriched.

### Protein interaction analysis

Analysis of differential gene-protein interaction networks was performed using the interaction relationship in the STRING protein interaction database (http://string-db.org/). Visual editing of differential gene-protein interaction network data files was performed by Cytoscape software version 3.5.1 (http://www.cytoscape.org/).

### qRT-PCR verification

To verify the accuracy of transcriptome sequencing data, nine candidate genes were randomly selected for qRT-PCR validation. The pituitary and hypothalamic tissue samples were obtained from 6 high-yield CDC hens and 6 low-yield CDC hens, and the egg production level classification criteria were consistent with those used for RNA-seq sequencing. After extracting total RNA, cDNA was synthetized using the total RNA reverse transcriptase kit (Takara, Dalian, China). Real-time PCR was performed on an ABI 7500 Fast Real-Time PCR System (Applied Biosystems, Foster City, CA, USA) using SYBR premix Ex TaqTM II (Takara). The optimised cycling conditions were denaturation at 94 °C for 5 min followed by 45 cycles of 94 °C for 15 s, and 55 °C for 15 s. Each sample was tested in triplicate. Relative expression was determined using the 2^−ΔΔCT^ method^45^, and beta-actin was used as the internal control for normalization of the results. All primer sequences are listed in Table 2.

**Table 2:** Primers used for qRT-PCR for the detection of DEGs.

Gene	Primer	Sequence (5′-3′)	Size(bp)	Reference sequence
*OVCH2*	Forward	AGCTTCAGAGGGAGGAAGGT	283	XM_025151046.1
	Reverse	CTTTCTGCCCGCACTTAGGA		
SLC7A10	Forward	TGTGTTACGCTGGAAGAAACC	239	XM_025154310.1
	Reverse	CACAGTGGCAGTGCTTACCTATT		
*VCAN*	Forward	AGGTGGGACCGCTACT	193	XM_419976.4
	Reverse	TTCTGTTGTCATGGCTGA		
*CATHL2*	Forward	GACACTCCCGAGATCAATCTACGC	163	FJ938358.1
	Reverse	CCCATTTATTCATTCAGCCAAAGC		
*GFRA4*	Forward	CTTCAGGCAGGTGAGTCCTG	248	NM_204991.1
	Reverse	TTCATGCCTTCCATCAGCGT		
*ESR1*	Forward	GCTTGCCGAGATCAGAGGAA	212	NM_205183.2
	Reverse	ATCTGGTGCAGCAGGGTAAC		
*LAMA1*	Forward	CACCCTGTGACTGTGCTCAT	271	NM_001199806.1
	Reverse	TCTGGGTAGTCCCTGTAGCC		
*CFD*	Forward	CCCACAAACGGCTCTACGACG	112	XM_003642860
	Reverse	TTCAGCTCCGCTTTCTCCTCC′		
*MANBAL1*	Forward	CGCTACGGCCTCTTCTTTG	177	XM_025142418
	Reverse	TTCCTTCTTGGCTTTCTTGCT		
beta-actin	Forward	CTGTGCCCATCTATGAAGGCTA	139	NM_205518
	Reverse	ATTTCTCTCTCGGCTGTGGTG		

## Supplementary information

Supplementary Information
