# Supplementary material for: Hypothalamic and pituitary transcriptome profiling using RNA-sequencing in high-yielding and low-yielding laying hens
Source: Sci Rep. 2019 Jul 16;9:10285. doi: 10.1038/s41598-019-46807-3 (PMC6635495; doi:10.1038/s41598-019-46807-3)
Supplement: Supplementary file 1 — Supplementary Information [file 41598_2019_46807_MOESM1_ESM.pdf]

Supplementary Information

Hypothalamic and pituitary transcriptome profiling using RNA-sequencing in high-yielding and low-yielding laying hens.

Chunqiang Wang, Wei Ma*

College of Animal Science and Veterinary Medicine, Jinzhou Medical University, Jinzhou, Liaoning, 121001, P. R. of China

* Corresponding author. E-mail address: mwworld@163.com.

S 1. RNA-seq data statistics and summary

Sample name	Raw reads	Clean reads	clean bases	Error rate (%)	Q20 (%)	Q30 (%)	GC content (%)	Total mapped	Multiple mapped	Uniquely mapped	reads to Exons
LP_1	68317482	66328810	9.95G	0.03	96.79	92.07	52.97	57680205 (86.96%)	2096037 (3.16%)	55584168 (83.8%)	66.60%
LP_2	72359294	69806998	10.47G	0.03	96.77	92.24	52.56	60386063 (86.5%)	2205690 (3.16%)	58180373 (83.34%)	62.10%
LP_3	63889132	62116040	9.32G	0.03	96.78	92.08	52.49	53666920 (86.4%)	1934202 (3.11%)	51732718 (83.28%)	64.40%
LH_1	79744218	77900688	11.69G	0.03	96.82	91.94	51.92	68487901 (87.92%)	2619234 (3.36%)	65868667 (84.55%)	69.50%
LH_2	65534460	62447384	9.37G	0.03	97.04	92.62	52.07	54556009 (87.36%)	2096260 (3.36%)	52459749 (84.01%)	66.60%
LH_3	79835258	78021862	11.7G	0.03	96.62	91.6	53.07	67701041 (86.77%)	2587685 (3.32%)	65113356 (83.46%)	68.20%
NP_1	64341034	61029484	9.15G	0.03	96.99	92.51	51.52	52964336 (86.78%)	2026556 (3.32%)	50937780 (83.46%)	64.10%
NP_2	69054884	67531462	10.13G	0.03	96.79	91.98	52.95	59190144 (87.65%)	2215613 (3.28%)	56974531 (84.37%)	65.20%
NP_3	62110264	60924682	9.14G	0.03	96.85	92.17	53.54	52608969 (86.35%)	1902904 (3.12%)	50706065 (83.23%)	64.60%
NH_1	76681332	74134976	11.12G	0.03	96.64	91.86	52.74	64570880 (87.1%)	2549583 (3.44%)	62021297 (83.66%)	70.70%
NH_2	49332078	46982632	7.05G	0.03	96.18	90.76	51.60	41205550 (87.7%)	1567425 (3.34%)	39638125 (84.37%)	67.10%
NH_3	73617140	71760796	10.76G	0.03	96.71	91.90	53.57	61408353 (85.57%)	2390137 (3.33%)	59018216 (82.24%)	66.90%

S 2. List of the whole DEGs

group	Gene_id	readcount_NP	readcount_LP	log2FoldChange	pval	padj	Gene name
NP vs LP	ENSGALG00000000293	487.5134113	142.606793	1.7734	2.90E-08	7.36E-05	-
	ENSGALG00000003053	5250.601495	10603.64015	-1.014	1.55E-05	0.013173	PRDX6
	ENSGALG00000003770	1076.824899	2234.866655	-1.0534	4.47E-06	0.0046133	ANXA2
	ENSGALG00000004729	54.87640492	196.8159733	-1.8426	2.71E-05	0.020767	SLC7A10
	ENSGALG00000005318	197.5145727	1961.573084	-3.312	6.41E-06	0.0058285	OVCH2
	ENSGALG00000009693	1594.810295	705.4449769	1.1768	9.67E-07	0.0016385	CPXM2
	ENSGALG00000012367	962.5484805	1830.466329	-0.92728	7.49E-05	0.045579	TRIM9
	ENSGALG00000012973	1652.749372	844.1350303	0.96932	2.35E-05	0.018623	ESR1
	ENSGALG00000014201	1356.312963	472.1222542	1.5225	3.10E-08	7.36E-05	PARVB
	ENSGALG00000014615	482.2527029	132.3755069	1.8652	5.56E-09	1.88E-05	LAMA1
	ENSGALG00000015193	1775.176393	6180.070946	-1.7997	6.63E-06	0.0058285	CCDC80
	ENSGALG00000015624	572.0225086	4873.409901	-3.0908	6.29E-05	0.039279	VCAN
	ENSGALG00000016457	4626.672823	2528.307566	0.8718	5.10E-05	0.033619	TRIB2
	ENSGALG00000016483	0.665182141	310.8060666	-8.868	1.22E-09	5.79E-06	-
	ENSGALG00000016785	941.9947138	405.567986	1.2158	2.41E-06	0.0031767	IL1RL1
	ENSGALG00000017040	213.6088279	61.4354577	1.7978	2.28E-05	0.018623	-
	ENSGALG00000019696	42.60450566	276.4344255	-2.6979	2.17E-09	8.58E-06	CATHL2
	ENSGALG00000026736	567.3547897	241.5348596	1.232	3.28E-05	0.0236	OGN
	ENSGALG00000027184	342.0645108	116.3626191	1.5556	5.84E-06	0.0057769	OLFML1
	ENSGALG00000032506	2139.448918	982.3847372	1.1229	1.25E-06	0.00186	GEM
	ENSGALG00000034150	57.05120496	472.0489374	-3.0486	1.58E-17	3.76E-13	-
	ENSGALG00000035131	229.294779	757.3197679	-1.7237	4.06E-06	0.0043779	-
	ENSGALG00000035631	636.06216	1622.600175	-1.3511	1.45E-08	4.29E-05	-
	ENSGALG00000035927	1110.572587	557.2601331	0.99488	4.80E-05	0.032542	ST8SIA5

	ENSGALG00000036589	700.7840056	162.948394	2.1046	3.34E-13	3.97E-09	GFRA4
	ENSGALG00000038433	950.2022047	352.444424	1.4308	7.89E-08	0.00017026	SPON2
	ENSGALG00000040114	1624.413015	3409.450087	-1.0696	3.56E-05	0.024825	PTPRZ1
	ENSGALG00000040832	26251.99862	8109.07813	1.6948	3.90E-10	3.08E-06	CFD
	ENSGALG00000041419	5766.779895	10699.47568	-0.8917	2.87E-05	0.021289	-
	ENSGALG00000042984	164.4653895	529.0147847	-1.6855	3.25E-06	0.0038548	-
	Novel00383	2687.366862	1301.813652	1.0457	2.97E-06	0.0037063	--
	Novel00399	534.7113643	238.2751673	1.1661	5.86E-05	0.037594	--
	Novel00460	32.67293273	180.982914	-2.4697	2.19E-07	0.00043362	--
	Novel00477	67.32980378	242.4592474	-1.8484	3.49E-06	0.0039457	--
	Novel00981	208.7626419	29.9466076	2.8014	5.80E-10	3.44E-06	-//-
	Novel01245	227.1573747	56.77377084	2.0004	1.25E-06	0.00186	-//-
	Novel01381	139.5915603	24.04354902	2.5375	1.68E-06	0.00235	--
	Novel02225	200.9520779	794.0595044	-1.9824	5.91E-07	0.0010794	-//-
	Novel02423	0	32.40993552		6.23E-06	0.0058285	-//-
	ENSGALG00000035631	644.2810926	1723.067316	-1.4192	2.29E-10	1.84E-06	-
	ENSGALG00000035660	65.65692291	226.3759527	-1.7857	4.86E-07	0.0019528	-//-
	ENSGALG00000041159	15.98602984	195.5383135	-3.6126	1.28E-12	1.54E-08	-
**NH vs LH**	Novel00002	360.3232343	129.1990689	1.4797	2.92E-06	0.010061	--
	Novel01245	598.9718272	97.08931575	2.6251	7.08E-20	1.71E-15	-//-
	Novel01887	766.304228	316.7715886	1.2745	2.96E-07	0.0014254	-//-
	Novel02735	200.9449235	30.17346296	2.7354	1.83E-08	0.00011033	-//-
